# Neutrophil-to-Lymphocyte Ratio Predicts Restenosis After Drug-Coated Balloon Therapy for Femoropopliteal Artery Lesions: A Retrospective Study

**DOI:** 10.3389/fcvm.2022.868656

**Published:** 2022-07-14

**Authors:** Zhihong Wang, Lei Sheng, Hongbin Gu, Fan Yang, Huajie Xie, Mingfei Li

**Affiliations:** Department of Vascular Intervention, People’s Liberation Army Strategic Support Force Characteristic Medical Center, Beijing, China

**Keywords:** neutrophils, lymphocytes, drug-coated balloon, femoropopliteal artery disease, restenosis

## Abstract

**Background:**

Peripheral artery disease (PAD) is a common atherosclerotic vascular disease. The use of drug-coated balloon (DCB) for the treatment of femoropopliteal artery disease has gradually increased. A certain percentage of patients developed target lesion restenosis after DCB treatment of the femoral popliteal artery. The neutrophil-to-lymphocyte ratio (NLR) is closely related to the level of inflammatory activity and has predictive value for atherosclerotic vascular disease. This study aimed to analyze the relationship between NLR and 1-year restenosis after DCB for femoropopliteal artery disease.

**Methods:**

Patients with femoropopliteal artery disease who were treated with DCBs at our hospital from May 2016 to December 2020 were retrospectively included. Baseline data during the patient’s first hospital stay and data during follow-up were collected. Demographic data, laboratory test results, lesion examination results, and major adverse events during the follow-up period were collected. Logistic regression was used to analyze the factors associated with restenosis after DCB.

**Results:**

A total of 117 patients were included. During 1-year follow-up, 19 cases (16.2%) of restenosis were detected. Five of these patients (4.3% of total included patients) were readmitted for symptomatic ischemia. No deaths or amputations occurred. Baseline NLR in patients with restenosis was higher than that in patients without restenosis (2.4 (2.1, 3.4) vs. 1.8 (1.3, 2.3), *P* < 0.001). Logistic univariate and multivariate analysis showed that baseline hs-CRP level (OR = 1.10, 95%CI: 1.05–1.34), lesion length (OR = 1.04, 95%CI: 1.02–1.27), use of rivaroxaban (OR = 1.08, 95%CI: 1.05–1.39), NLR (OR = 1.47, 95%CI: 1.13–2.48), LDL-C level (OR = 1.25, 95%CI: 1.05–1.52), and diabetes (OR = 1.25, 95%CI: 1.05–1.52) = 1.18, 95%CI: 1.06–1.66) were predictors of restenosis.

**Conclusion:**

Baseline NLR before DCB can predict the risk of restenosis after surgery.

## Introduction

Peripheral artery disease (PAD) is also a common atherosclerotic vascular disease similar to coronary atherosclerotic disease. It is relatively rare in young patients under the age of 50, but the incidence increases rapidly with age, which reaches 20% in people in their 80 s (1, 2). The Global Peripheral Artery Disease Study in 2013 showed for the first time that around 2 million PAD patients were reported worldwide (3); among them, lower extremity arterial disease was the most (1). In lower extremity arterial disease, the femoropopliteal artery has a larger lumen and is often treated with intervention combined with drugs; meanwhile, the distal small artery intervention is more difficult, and drug therapy or surgery is mainly used (4). Benedetto et al. demonstrated that inframalleolar bypass resulted in good patency rates, limb salvage and overall survival (5). Currently, two main methods are used for interventional treatment of femoropopliteal artery disease, namely, stent implantation and balloon dilation (4) followed by strict medical therapy, including long-term antiplatelet therapy and statin (6). In recent years, drug-coated balloon (DCB) has been gradually applied in the treatment of femoropopliteal artery disease, especially restenosis after stent implantation (7–10). With the increasing application of DCB, the number of direct uses of DCB to treat femoropopliteal artery disease has also increased significantly (11–13). However, even in the era of interventional therapy combined with intensive drug therapy, some patients still develop target lesion restenosis after interventional therapy, especially in patients undergoing stent implantation (14, 15). In a study using balloon dilation for femoropopliteal artery disease, the rate of restenosis at 6 months after surgery was 33% (16). In a study with long-term follow-up, the results showed that femoropopliteal artery lesions with different TASC II classifications had different patency rates at 5-year postoperative follow-up after endovascular therapy, with type D lesions generally having the lowest rate of only 34% (17). A certain percentage of patients developed target lesion restenosis after DCB treatment of the femoropopliteal artery (18, 19), and studies even found that DCB treatment of femoropopliteal artery disease increases the risk of death in patients (20). Therefore, screening the predictive factors of restenosis after DCB treatment for femoropopliteal artery disease has important clinical significance.

At present, a large number of studies believe that restenosis is closely related to the local inflammatory activity of target lesions (21, 22). Although drugs coated on the surface of the balloon, such as paclitaxel, have the effect of suppressing inflammation (23), but maintaining a sustained effect is difficult. When the local drug metabolites and completely disappears and the inflammatory activity at the lesion site increases, local smooth muscle cells migrate and proliferate, which result in the formation of stenosis again (24). Previous studies have found that the neutrophil-to-lymphocyte ratio (NLR) is closely related to the level of inflammatory activity and has some predictive value for various diseases or adverse events, especially in atherosclerotic vascular disease (25–27). On the basis of these findings, this study aimed to retrospectively analyze the predictive value of baseline NLR for postoperative restenosis with DCB in the treatment of femoropopliteal artery disease.

### Study Population

This study was retrospective. Patients with femoropopliteal artery disease who were treated with DCBs at our hospital from May 2016 to December 2020 were included. Inclusion criteria were as follows: patients with obvious lower extremity arterial ischemia symptoms and Rutherford grade of 3–5; patients with femoropopliteal artery disease who received interventional therapy for the first time; patients who received DCB therapy; patients aged ≥18 years; patients with complete clinical data; patients who completed 1-year scheduled follow-up; patients who met the diagnostic criteria for restenosis; patients who signed the informed consent. Exclusion criteria were as follows: patients with femoropopliteal artery occlusion and stenosis caused by vasculitis secondary to rheumatic diseases; patients with combined malignant tumors; patients not insisting on taking antithrombotic drugs as prescribed by the doctor after operation. All patients signed informed consent. This study was approved by the Ethics Committee of the People’s Liberation Army Strategic Support Force Characteristic Medical Center.

### Interventions

All patients underwent color Doppler ultrasonography and CT angiography before DCB surgery to further clarify the characteristics of the lesions. DCB interventional therapy was as follows: the patient was placed in a supine position and sterilized with iodophor. Local anesthesia was performed with 2% lidocaine at the puncture site, and then, the Seldinger technique was used to puncture and implant the sheath, and the guide wire was inserted through the sheath to reach the site of target lesion. The patient was anticoagulated intraoperatively with 50–100 IU/kg heparin, and the activated coagulation time was maintained at 250 s. A 0.035- or 0.018-inch guide wire was used to pass through the target lesion. After angiography confirmed that the distal end was in the true lumen of the blood vessel, a normal balloon with a diameter of 4–5 mm was used for pre-dilation for 2 min and then withdrawn. When retraction or dissection formation did not exist, the target lesion was expanded with a paclitaxel-coated balloon of the same diameter as the ordinary balloon for 3–4 mins. The dose density of paclitaxel was 3.3 mg/mm^2^ (total weight of drug per unit of balloon surface area). The operation was defined as successful when the repeated angiography showed that the residual stenosis of the lumen was less than 30%. All patients took aspirin of 100 mg once a day and clopidogrel of 75 mg once a day before intervention (for at least 3 days) and during 1-year follow-up after intervention to reduce adverse events related with intervention and other high risk of cardiovascular diseases at the same time. Rivaroxaban of 2.5 mg twice a day instead of clopidogrel after surgery was prescribed in some patients for at least 1 year. Other drugs were administrated according to the patient’s comorbidities.

### Follow-Up

All patients received regular outpatient follow-up after surgery. The follow-up time was 7 days, 1, 3, 6, and 12 months after surgery, and an in-hospital angiography was reviewed at the 12-month follow-up which was a regular care for these patients. The contents of each follow-up mainly included symptoms, signs, and color Doppler ultrasonography. Restenosis was defined as >50% lumen stenosis or a peak systolic velocity ratio >2.5 at the target lesion on color Doppler ultrasonography. During the follow-up period, patients can go to the outpatient clinic at any time according to the actual situation and be hospitalized if necessary for further examination.

### Data Collection

Baseline data during the patient’s first hospital stay and data during follow-up were collected. Baseline data were information before DCB surgery, including demographic data, laboratory test results, and lesion examination results. The information collected during the follow-up period mainly included: major adverse events, including all-cause death, amputation of the lower extremity where the target lesion was located, revascularization treatment, and the results of the 12-month angiographic review.

Statistical analysis was performed using SPSS software (version 22.0, IBM, Chicago, IL, United States). When continuous variables conform to a normal distribution, they were expressed as the mean ± standard deviation, and the comparison between groups was by student *t*-test. The data were expressed as n (%), and the chi-square test or Fisher’s exact test was used for comparison between groups. Logistic regression was used to analyze the relationship between each factor and target lesion restenosis. *P* < 0.05 indicated that the difference was statistically significant.

## Results

### Baseline Characteristics

According to the inclusion and exclusion criteria, 117 patients were included. Age was 48–77 years, with a median age of 67.0 (59.0, 71.0). A total of 77 males (65.8%) and 40 females (34.2%) were enrolled (Table 1).

**TABLE 1:** Patient baseline information.

Characteristics	Value
Age (yrs)	67.0 (59.0, 71.0)
Male (n,%)	77 (65.8)
Smoke (n,%)	12 (10.3)
Alcohol (n,%)	11 (9.4)
Hypertension (n,%)	48 (41.0)
Diabetes (n,%)	76 (65.0)
CHD (n,%)	11 (9.4)
Glucose (mmol/L)	5.3 (4.8, 6.0)
TC (mmol/L)	5.5 (4.7, 6.1)
LDL-C (mmol/L)	4.4 (3.7, 5.1)
TG (mmol/L)	2.1 (1.9, 2.3)
WBC (× 10^9^/L)	6.0 ± 0.9
NEU (× 10^9^/L)	3.4 ± 0.5
LYM (× 10^9^/L)	1.7 (1.5, 2.4)
NLR	1.9 (1.3, 2.4)
RBC (× 10^12^/L)	4.8 (4.6, 5.0)
Hb (g/L)	146.0 (143.0, 148.5)
PLT (× 10^9^/L)	169.0 (152.0, 212.0)
ALT (U/L)	30.2 ± 8.0
Cr (μmol/L)	69.2 ± 12.8
CRP (mg/L)	3.5 (2.4, 4.6)
Length (mm)	80.0 (76.0, 84.0)
Duration (years)	1.5 (0.9, 2.2)
Rivaroxaban (n,%)	13 (11.1)
Aspirin before intervention (n,%)	
Clopidogrel before intervention (n,%)	
Statin before intervention (n,%)	
Rutherford stage	
3	1 (0.9)
4	61 (52.1)
5	41 (35.0)
6	14 (12.0)
Total occlusion (n,%)	72 (61.5)

*CHD, coronary heart disease; TC, total cholesterol; TG, triglyceride; WBC, white blood cell; NEU, neutrophil; LYM, lymphocyte; NLR, neutrophil lymphocyte ratio; RBC, red blood cell; Hb, hemoglobin; PLT, platelet; ALT, alanine transaminase; Cr, creatine; CRP, C-reaction protein.*

### One-Year Follow-Up Outcome

The follow-up time was 1–4.5 years with a median of 2.3 (1.4–3.1) years. In this study, only the 1-year follow-up results of each patient after DCB were counted. During 1-year follow-up, 19 (16.2%) restenosis were detected. Among them, 5 patients (4.3% of total included patients) were re-hospitalized due to symptomatic ischemia and underwent target lesion revascularization treatment for 4–10 months after the first postoperative DCB (Table 2). No deaths and no major bleeding occurred. Only one case had mild gingival bleeding, and the bleeding stopped spontaneously with no special treatment.

**TABLE 2:** 1-year follow-up outcomes after DCB.

Outcomes	n	%
Restenosis	19	16.2
TLR	5	4.3
Minor bleeding	1	0.9
Major bleeding	0	0
All-cause death	0	0

### Predictive Factors for Restenosis 1-Year After Drug-Coated Balloon Treatment for Femoral–Popliteal Artery Lesions

The patients were divided into restenosis and non-restenosis groups according to the occurrence of restenosis. The baseline data comparison of the two groups is listed in Table 3. Differences existed in several indicators between the two groups of patients, including the ratio of diabetic patients, total cholesterol level, low-density lipoprotein cholesterol ester (LDL-C), white blood cell count, lymphocyte count, NLR, creatinine level, hs-CRP level, lesion length, disease duration, and rate of rivaroxaban use. Further logistic univariate and multivariate analysis showed that baseline hs-CRP level, lesion length, use of rivaroxaban, NLR, LDL-C levels, and diabetes mellitus were predictors of restenosis (Table 4).

**TABLE 3:** Baseline data comparison of patients with restenosis and non-restenosis.

Characteristics	Restenosis (*n* = 19)	Non-restenosis (*n* = 98)	Statistical value	*P*-value
Age (yrs)	69.0 (64.0, 71.5)	66.0 (59.0, 66.0)	1.279	0.201
Male (n,%)	13 (68.4)	64 (65.3)	0.068	0.793
Smoke (n,%)	3 (15.8)	12 (12.2)	0.002	0.962
Alcohol (n,%)	3 (15.8)	11 (11.1)	0.031	0.861
Hypertension (n,%)	8 (42.1)	40 (40.8)	0.011	0.917
Diabetes (n,%)	17 (89.5)	59 (60.2)	5.990	0.014
CHD (n,%)	2 (10.5)	9 (9.2)	0.061	0.806
Glucose (mmol/L)	5.4 (4.6, 7.5)	5.3 (4.8, 5.9)	0.624	0.532
TC (mmol/L)	5.8 (5.5, 6.2)	5.4 (4.5, 6.1)	2.646	0.008
LDL-C (mmol/L)	4.5 (4.2, 5.1)	4.4 (3.7, 5.1)	3.289	0.001
TG (mmol/L)	2.1 (1.9, 2.3)	2.2 (1.9, 2.3)	1.212	0.226
WBC (× 10^9^/L)	5.1 ± 0.4	6.1 ± 0.9	4.760	<0.001
NEU (× 10^9^/L)	3.3 ± 0.4	3.4 ± 0.5	1.164	0.247
LYM (× 10^9^/L)	1.2 (1.1, 1.5)	1.8 (1.5, 2.7)	5.066	<0.001
NLR	2.4 (2.1, 3.4)	1.8 (1.3, 2.3)	3.880	<0.001
RBC (× 10^12^/L)	4.7 (4.6, 5.0)	4.8 (4.6, 5.0)	0.170	0.865
Hb (g/L)	145.1 (142.5, 148.4)	146.1 (143.2, 148.5)	0.676	0.499
PLT (× 10^9^/L)	201.0 (150.5, 220.5)	167.5 (152.3, 209.3)	0.695	0.487
ALT (U/L)	26.9 ± 6.4	30.8 ± 8.2	1.937	0.055
Cr (μmol/L)	75.8 ± 11.6	68.0 ± 12.7	2.489	0.014
Hs-CRP (mg/L)	6.4 (5.5, 7.1)	3.3 (2.1, 4.1)	4.866	<0.001
Length (mm)	86.0 (81.5, 86.5)	79.0 (76.0, 82.0)	3.754	<0.001
Rutherford stage			5.276	0.153
3	1 (5.3)	0 (0)		
4	10 (52.6)	51 (52.1)		
5	6 (31.6)	35 (35.7)		
6	2 (10.5)	12 (12.2)		
Total occlusion (n,%)	11 (57.9)	61 (62.2)	0.127	0.721
Duration (years)	2.7 (1.9, 4.0)	1.3 (0.8, 1.8)	4.730	<0.001
Rivaroxaban (n,%)	2 (10.5)	37 (37.8)	5.310	0.021

*CHD, coronary heart disease; TC, total cholesterol; TG, triglyceride; WBC, white blood cell; NEU, neutrophil; LYM, lymphocyte; NLR, neutrophil lymphocyte ratio; RBC, red blood cell; Hb, hemoglobin; PLT, platelet; ALT, alanine transaminase; Cr, creatine; CRP, C-reaction protein.*

**TABLE 4:** Predictive factors for 1-year restenosis after DCB for femoropopliteal artery disease.

Predictors	Univariate Analysis			Multivariate Analysis		
	OR	95%CI	*P*-value	OR	95%CI	*P*-value
hs-CRP	1.14	1.06–1.37	0.025	1.10	1.05–1.34	0.033
Lesion length	1.08	1.03–1.24	0.031	1.04	1.02–1.27	0.041
Rivaroxaban	1.12	1.05–1.41	0.022	1.08	1.05–1.39	0.036
NLR	1.56	1.18–2.53	0.009	1.47	1.13–2.48	0.010
LDL-C	1.29	1.09–1.63	0.016	1.25	1.05–1.52	0.024
Diabetes	1.21	1.10–1.74	0.011	1.18	1.06–1.66	0.019

## Discussion

This study retrospectively analyzed the clinical and follow-up data of patients with femoropopliteal artery disease treated with DCB. The results showed that the incidence of restenosis in the 1-year follow-up after DCB treatment of femoropopliteal artery disease was 16.2%. Preoperative baseline hs-CRP level, LDL-C level, NLR, diabetes, lesion length, and long-term postoperative rivaroxaban were predictors of restenosis within 1 year after intervention. In the present study, 5 (4.3% of total included patients) target lesion revascularization (TLR) cases occurred during the 1-year follow-up, but no deaths occurred.

Peripheral artery disease often leads to claudication due to ischemia and even necrosis of the limbs in the blood supply area of the diseased blood vessels. In severe cases with PAD, amputation is required and PAD is associated with an increased risk of death. Effective interventional therapy can rapidly improve blood supply to the limbs and relieve or even eliminate pain. However, after a long-term clinical study, the researchers found that the incidence of restenosis after interventional therapy is higher regardless whether it is simple balloon dilation, or implantation of bare metal stents or drug-eluting stents. Dealing with restenosis is difficult once it occurs. Therefore, earlier studies focused on predictors associated with in-stent restenosis. DCB was initially used in the treatment of in-stent restenosis and achieved good results. Since then, the direct use of DCB to treat PAD, especially femoropopliteal artery disease, has become more widespread. Similarly, target lesion patency or restenosis after DCB has also received increasing attention from clinicians. In a retrospective study (18), Zhen et al. found that the 6-month target lesion patency rate (6-month primary patency) after DCB treatment for femoropopliteal artery disease was 77.3%, while the 6-month target lesion patency after uncoated balloon (UCB) treatment was only 53.2% (*P* = 0.011). The definition of patency in the previous study is <50% lumen stenosis and the ratio of peak systolic velocity >2.4, which is consistent with the definition of restenosis in this study (18). Therefore, the incidence of restenosis at 6 months after DCB in this study was approximately 22.7%, which was higher than the 16.2% in this study, which was not statistically different when patient heterogeneity was ignored (18). This study further analyzed the predictive value of preoperative and postoperative NLR on target lesion patency at 6 months after balloon dilation. The results showed that preoperative NLR had no predictive effect, but the rate was higher when the postoperative NLR was lower (18). The results of our study were inconsistent with those of Zhen et al. possibly because the subjects included in the two studies were inconsistent. Only patients treated with DCB were included in this study, whereas more than half (62/108) of the patients in the study by Zhen et al. were treated with UCB (18). Some factors closely related to atherosclerosis were also included in this study, including hs-CRP, LDL-C, and smoking and drinking. Furthermore, some of the patients in this study were taking rivaroxaban, which may have potential to reduce the incidence pf restenosis (28). The results of this study also showed that long-term low-dose rivaroxaban may reduce the risk of restenosis after surgery. We believe that this may depends on the antithrombotic effect of rivaroxaban. In the Ranger II SFA study published in 2021, the rate of TLR during the 12-month follow-up period was 5.5% (14/256), which was close to the results of this study (29). In another study, Yoshioka analyzed factors associated with paclitaxel-eluting stent (PES) and DCB-related restenosis for 1-year after femoropopliteal artery surgery (14). The results showed that the factors associated with restenosis after PES implantation included clinical frailty scale, gender, distal vessel diameter, body mass index, and age; meanwhile, those associated with restenosis after DCB dilation included peripheral artery calcification scoring system classification, post-dissection severity, vessel diameter (proximal and distal), and lesion length, and these results were significantly inconsistent with this study (14). Possible reasons included, first, the study excluded laboratory indicators (such as CRP and LDL-C); second, the rate of hypertensive patients (80.8%) in this study was high; third, the sample size of patients that received DCB treatment in this study was smaller than this study (86 vs. 117). As mentioned above, atherosclerotic disease is an inflammatory disease in nature, and the systemic inflammatory state and the local inflammatory activity of the lesion are related to the severity of the lesion and its response to treatment. Therefore, many studies have included indicators reflecting inflammatory activity, such as CRP and neutrophils, when analyzing atherosclerosis-related adverse events (30, 31). Similarly, atherosclerotic disease is closely related to LDL-C, and substantial evidence suggests that LDL-C is a risk factor for multiple atherosclerotic diseases and an independent predictor of many adverse events (32–34). NLR is an indicator of inflammatory activity that has received increasing attention in recent years. High NLR increases the risk of unstable plaque and severe vascular stenosis, as well as the risk of major adverse cardiovascular events, including metabolic syndrome, sepsis, malignancy, and poor patient outcomes in patients with coronary heart disease (27). NLR was also investigated in patients with lower extremity peripheral artery disease (PAD). Teperman et al. found that elevated NLR was an indicator of severe multi-level PAD (35). In another study by Taurino et al., the authors found that preoperative NLR level was related to acute limb ischemia outcomes in unselected population (36). Importantly, NLR is easy to obtain and can be directly calculated based on the data of blood cell counts in routine laboratory tests of patients, without increasing the medical expenses of patients and the workload of doctors. Baseline NLR reflects the patient’s preoperative baseline inflammatory activity. Although according to current clinical guidelines, many patients received moderate to high-intensity statin therapy (6, 37). However, completely inhibiting the local inflammatory activity of target lesions is difficult. Therefore, the level of basal inflammatory activity may be related to the occurrence of target lesions and their response to therapy. In the short term after DCB treatment, the local inflammatory activity in the target lesion was inhibited by the drug. Furthermore, multiple factors can influence the outcome of NLR after surgery, including stress (38) and bacterial infection. At the same time, additional blood test for NLR calculation after surgery also increases the patient’s medical costs and may reduce patient compliance.

This study also observed hat diabetes was a risk factor for restenosis after interventional atherosclerotic vascular stenosis, which was consistent with the results of many studies (39, 40). However, some studies suggested that diabetes was not a risk factor for restenosis after lower extremity arterial intervention, but it increases the risk of death and claudication during postoperative follow-up (41). Our study also found that lesion length was associated with restenosis after DCB. Some previous studies have also found that increased lesion length also increases the risk of restenosis (42). The use of rivaroxaban or other anticoagulant drugs in the treatment of lower extremity arterial disease after intervention has also gradually increased (43). Some studies have shown that long-term use of low-dose rivaroxaban after arterial interventional therapy can reduce the risk of adverse events (44) and further systematic review results also demonstrated that rivaroxaban in combination with aspirin reduces the risk of postoperative revascularization in patients with PAD (45).

This study also has some limitations. First, this study has some limitations common to retrospective analysis studies. For example, some patients were excluded in the study due to incomplete data, which resulted in a small sample size or some potential predictive factors that could not be analyzed. Second, this study is a single-center study, and some case selection bias may exist. Third, the baseline level detection of laboratory indicators in this study were all preoperative results, but the time points were not uniform, and certain differences existed in the detection conditions. Fourth, this study failed to analyze the preoperative infection status of patients, which directly affects the results of neutrophil counts. In view of the abovementioned problems, prospective studies can be conducted in the future to control most of the factors as much as possible and reduce the variation.

## Conclusion

Baseline NLR before DCB therapy in patients with femoropopliteal artery disease can predict the risk of restenosis during 1-year follow-up. Further prospective, multicenter studies are needed to confirm the results of this study.

## Data Availability Statement

The original contributions presented in this study are included in the article/supplementary material, further inquiries can be directed to the corresponding author.

## Ethics Statement

The studies involving human participants were reviewed and approved by Ethics Committee of the People’s Liberation Army Strategic Support Force Characteristic Medical Center. The patients/participants provided their written informed consent to participate in this study.

## Author Contributions

ZW and LS: conception and design. ZW: administrative support. LS: provision of study materials or patients. ZW, LS, and HG: collection and assembly of data. ZW, FY, HX, and ML: data analysis and interpretation.

## Conflict of Interest

The authors declare that the research was conducted in the absence of any commercial or financial relationships that could be construed as a potential conflict of interest.

## Publisher’s Note

All claims expressed in this article are solely those of the authors and do not necessarily represent those of their affiliated organizations, or those of the publisher, the editors and the reviewers. Any product that may be evaluated in this article, or claim that may be made by its manufacturer, is not guaranteed or endorsed by the publisher.

## Abbreviations

PAD: Peripheral artery disease
DCB: drug-coated balloon
NLR: neutrophil-to-lymphocyte ratio
OR: odd ratio
CHD: coronary heart disease
TC: total cholesterol
TG: triglyceride
WBC: white blood cell
NEU: neutrophil
LYM: lymphocyte
RBC: red blood cell
Hb: hemoglobin
PLT: platelet
ALT: alanine transaminase
Cr: creatine
CRP: C-reaction protein.
